# Olfactory Sensitivity for Six Predator Odorants in CD-1 Mice, Human Subjects, and Spider Monkeys

**DOI:** 10.1371/journal.pone.0080621

**Published:** 2013-11-20

**Authors:** Amir Sarrafchi, Anna M. E. Odhammer, Laura Teresa Hernandez Salazar, Matthias Laska

**Affiliations:** 1 Department of Physics, Chemistry and Biology, Linköping University, Linköping, Sweden; 2 Instituto de Neuro-Etologia, Universidad Veracruzana, Xalapa, Veracruz, Mexico; Barnard College, Columbia University, United States of America

## Abstract

Using a conditioning paradigm, we assessed the olfactory sensitivity of six CD-1 mice *(Mus musculus)* for six sulfur-containing odorants known to be components of the odors of natural predators of the mouse. With all six odorants, the mice discriminated concentrations <0.1 ppm (parts per million) from the solvent, and with five of the six odorants the best-scoring animals were even able to detect concentrations <1 ppt (parts per trillion). Four female spider monkeys *(Ateles geoffroyi)* and twelve human subjects *(Homo sapiens)* tested in parallel were found to detect the same six odorants at concentrations <0.01 ppm, and with four of the six odorants the best-scoring animals and subjects even detected concentrations <10 ppt. With all three species, the threshold values obtained here are generally lower than (or in the lower range of) those reported for other chemical classes tested previously, suggesting that sulfur-containing odorants may play a special role in olfaction. Across-species comparisons showed that the mice were significantly more sensitive than the human subjects and the spider monkeys with four of the six predator odorants. However, the human subjects were significantly more sensitive than the mice with the remaining two odorants. Human subjects and spider monkeys significantly differed in their sensitivity with only two of the six odorants. These comparisons lend further support to the notion that the number of functional olfactory receptor genes or the relative or absolute size of the olfactory bulbs are poor predictors of a species’ olfactory sensitivity. Analysis of odor structure**–**activity relationships showed that in both mice and human subjects the type of alkyl rest attached to a thietane and the type of oxygen moiety attached to a thiol significantly affected olfactory sensitivity.

## Introduction

Prey species display behavioral and sensory adaptations that allow them to recognize and avoid predators. For many mammalian prey species, this includes the ability to detect odors emitted by predators, and a number of studies have shown that predator odors elicit adaptive behavioral responses such as freezing or avoidance [1], [2]. Thus, predator odors act as kairomones, that is, as semiochemicals emitted by an organism (in this case: the predator) which mediates interspecific interactions in a way that benefits an individual of another species which perceives it (in this case: the prey), without benefiting the emitter [3]. Chemoanalytical studies found that the odor of urine, faeces and anal gland secretions of mammalian predators are characterized by volatile sulfur-containing metabolites that are products of their meat-eating and thus animal protein-rich diet [4], [5]. The odors produced by herbivorous mammals, in contrast, appear to largely lack such sulfur-containing compounds allowing prey species to efficiently distinguish between the odors of predator and non-predator species [6]–[9]. Interestingly, the presentation of sulfur-containing predator odorants as single compounds instead of as part of a complex mixture has been found to be sufficient to elicit repellent effects in a number of prey species [10]–[12], although some studies reported the complex mixture that predator-derived faecal or urine odors are composed of to be even more repellent compared to single compounds [13]. Despite the important role that predator odors play for many prey species, surprisingly little is known about the olfactory sensitivity of mammals for such sulfur-containing predator odorants.

It was therefore the aim of the present study to determine olfactory detection thresholds in mice for six sulfur-containing odorants known to be components of the odors of natural predators of the mouse. We decided to test spider monkeys and human subjects in parallel for the following reasons: 1. With all three species, the number of functional olfactory receptor genes as well as the absolute and the relative size of the olfactory bulbs are known, allowing us to assess the impact of these genetic and neuroanatomical features, respectively, on olfactory sensitivity. 2. In contrast to the mice, both primate species are unlikely to be natural prey species of the small-bodied carnivores known to be the source of the predator odorants tested here, allowing us to assess whether the behavioral relevance of the odorants (as indicators of the presence of predators) or their chemical nature (as sulfur-containing volatiles) affects olfactory sensitivity. 3. With all three species, olfactory detection thresholds have been determined in earlier studies with other sets of odorants, allowing us to assess whether mice, spider monkeys and humans are particularly sensitive to sulfur-containing odorants. 4. The fact that two pairs of the six predator odorants used here are structurally related to each other allowed us to assess the impact of molecular structural features on detectability and whether such effects, if they occur, are species-specific or general.

More specifically, we aimed at testing the following predictions:

1. Mice are more sensitive to odorants known to be components of the odors of natural predators of the mouse compared to human subjects and spider monkeys.

2. The number of functional olfactory receptor genes correlates positively with olfactory sensitivity for the odorants tested.

3. The relative and/or the absolute size of the olfactory bulbs correlate(s) positively with olfactory sensitivity for the odorants tested.

4. Mice, spider monkeys, and human subjects are more sensitive to the sulfur-containing odorants tested here compared to odorants lacking sulfur.

5. Molecular structural features of the sulfur-containing odorants tested here have a systematic effect on detectability.

## Materials and Methods

### Ethics statement

The animal experiments reported here comply with the *Guide for the Care and Use of Laboratory Animals* (National Institutes of Health Publication no. 86-23, revised 1985). The mouse experiments were performed according to a protocol approved by Linköping’s Animal Care and Use Committee (Linköpings djurförsöksetiska nämnd, protocol #69-09), and the spider monkey experiments were performed according to a protocol approved by the ethical board of the Federal Government of Mexico’s Secretariat of Environment and Natural Resources (SEMARNAT; Official permits no. 09/GS-2132/05/10). Both protocols included specific approval for the study reported here. The spider monkeys were kept in two outdoor enclosures of 6×4×4 m at the Field Station Pipiapan maintained by the Universidad Veracruzana (Mexico) and were thus exposed to natural environmental conditions concerning ambient temperature, relative humidity and light. Attached to the outdoor enclosures were single cages that could be closed by sliding doors to allow temporary separation of animals for individual testing. The animals were accustomed to this procedure and entered the single cages voluntarily upon calling their name. The enclosures were provided with climbing frames, ropes and swings and environmental enrichment was provided in the form of toys (e.g. balls and tyres) as well as branches and other manipulable parts of vegetation. The monkeys were fed fresh fruit and vegetables ad libitum. The amount of food offered daily to the animals was such that leftovers still were present on the floor the next morning. As the method employed in this study (a food-rewarded instrumental conditioning procedure) is based on the voluntary cooperation of the animals and works without any restraint or coercion, it was not necessary to take steps in order to alleviate suffering. None of the animals was sacrificed.

The human experiments comply with the declaration of Helsinki/Hong Kong. They were performed according to a protocol approved by the Institutional Review Board at the Department of Physics, Chemistry and Biology of Linköping University (protocol #14-10). All subjects were informed as to the aims of the study and provided written consent.

### Animals

Testing was carried out using six male CD-1 mice *(Mus musculus)* and four female spider monkeys *(Ateles geoffroyi)*. The rationale for choosing this outbred strain of mice was to use animals with a genetic background that is more similar to wild-type mice than that of inbred strains. Furthermore, data on olfactory detection thresholds for homologous series of aliphatic aldehydes [14] and carboxylic acids [15] as well as for structurally related aromatic aldehydes [16], alkylpyrazines [17], monoterpenes [18], “green odors” [19], and amino acids [20] were obtained in earlier studies using the same mouse strain. Four of the mice were 6 months old at the beginning of the study, and the remaining two mice were 18 months old. The rationale for choosing spider monkeys was that data on olfactory detection thresholds for homologous series of aliphatic esters [21], carboxylic acids [22], alcohols and aldehydes [23] as well as for thiols and indols [24] monoterpenes [18], monoterpene alcohols [25], steroids [26], [27], alkylpyrazines [17], aromatic aldehydes [28], “green odors” [19], and amino acids [20] were obtained in earlier studies using the same animals allowing us to compare their performance between the sulfur-containing odorants tested here and members of other chemical classes. The four spider monkeys were 6, 7, 11, and 14 years of age, respectively, at the beginning of the study. Maintenance of both species has been described in detail elsewhere (mice: [14]; spider monkeys: [29]).

### Human subjects

Twelve healthy, unpaid volunteers, six males and six females between 20 and 40 years of age, participated. The average age of the males was 29.3 years and that of the females was 30.3 years. None of the subjects had any history of olfactory dysfunction or suffered from an acute upper respiratory tract infection.

### Odorants

A set of six odorants was used: 2-propylthietane (CAS# 70678-49-8) has been found in the anal gland secretions of stoat *(Mustela erminea),* ferret *(Mustela putorius furo)*, Siberian weasel *(Mustela sibirica)*, and steppe polecat *(Mustela eversmanni)* [30], [31]. 2,2-dimethylthietane (CAS# 55022-72-5) has been found in the anal gland secretions of stoat *(Mustela erminea)*, ferret *(Mustela putorius furo)*, mink *(Mustela vison)*, Siberian weasel *(Mustela sibirica)*, and steppe polecat *(Mustela eversmanni)* [30], [31]. 3-mercapto-3-methylbutan-1-ol (CAS# 34300-94-2) has been found in cat *(Felis catus)* and bobcat *(Felis rufus)* urine [32], [33]. 3-mercapto-3-methylbutyl formate (CAS# 50746-10-6) has been found in cat *(Felis catus)* urine [32]. 3-methyl-1-butanethiol (CAS# 541-31-1) has been found in the anal gland secretions of striped skunk *(Mephitis mephitis)*, hooded skunk *(Mephitis macroura)*, and spotted skunk *(Spilogale putorius)* [34]. Methyl-2-phenylethyl sulfide (CAS# 5925-63-3) has been found in red fox *(Vulpes vulpes)* urine [35].

**Figure 1:**
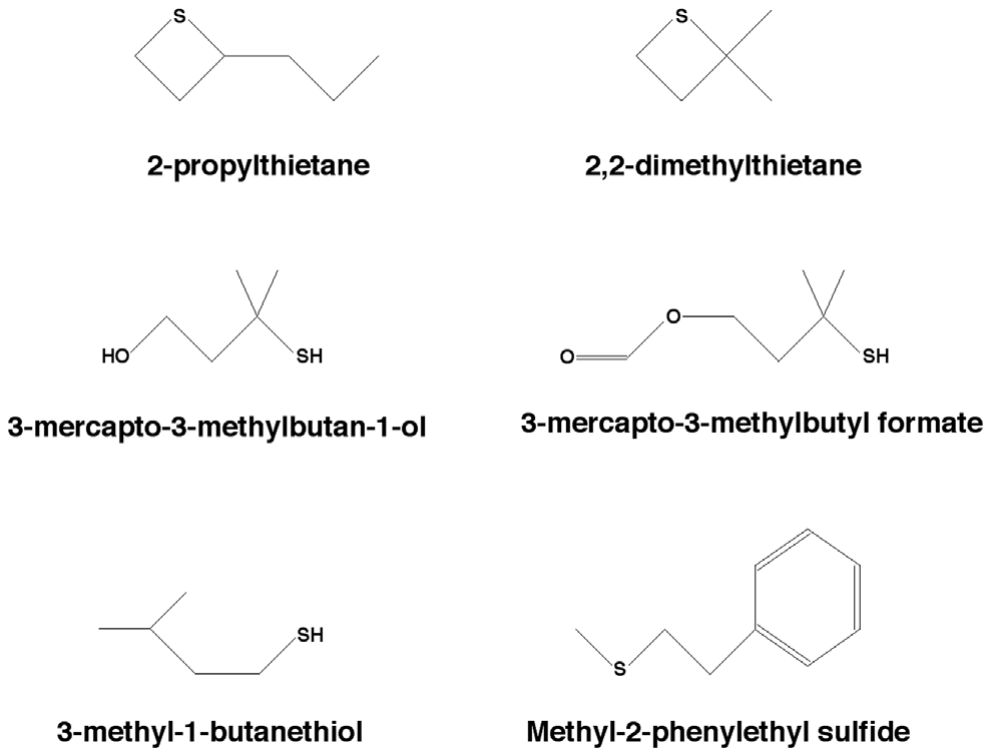
Chemical structure of the six sulfur-containing predator odorants used.

The rationale for choosing these substances was to assesss the sensitivity of the mice for sulfur-containing odorants known to be components of the odor of natural predators of the mouse. Previous studies have shown that the presentation of these odorants elicits avoidance responses in a variety of rodent prey species and thus that they indeed act as kairomones [6]-[9]. All substances were obtained from Sigma-Aldrich (St. Louis, MO) and had a nominal purity of at least 99%. They were diluted using near-odorless diethyl phthalate (CAS# 84-66-2) as the solvent. Gas phase concentrations for the headspace above the diluted odorants were calculated using published vapor pressure data [36] and corresponding formulae [37]. Figure 1 shows the molecular structure of the odorants.

### Behavioral test: mice

Olfactory sensitivity of the mice was assessed using an automated liquid-dilution olfactometer (Knosys, Tampa, FL) and an instrumental conditioning procedure which has been described in detail elsewhere [38]. Briefly, animals were trained to insert their snout into the odor sampling port of a test chamber. This triggered a 2 s presentation of either an odorant used as the rewarded stimulus (S+) or a blank (headspace of the solvent) used as the unrewarded stimulus (S–). Licking at a steel tube providing 2.5 µl of water reinforcement in response to presentation of the S+ served as the operant response. Forty such trials (20 S+ and 20 S– trials in pseudorandomized order) using the same concentration of a given S+ were conducted per animal and condition.

Olfactory detection thresholds were determined by testing the animals' ability to discriminate between increasing dilutions of an odorant used as S+, and the solvent alone used as S–. Starting with a gas phase concentration of 1 ppm (parts per million), each stimulus was successively presented in 10-fold dilution steps until an animal failed to significantly discriminate the odorant from the solvent. Subsequently, an intermediate concentration (0.5 log units between the lowest concentration that was detected above chance and the first concentration that was not) was tested in order to determine the threshold value more exactly.

### Behavioral test: spider monkeys

Olfactory sensitivity of the spider monkeys was assessed using a food-rewarded instrumental conditioning procedure which has been described in detail elsewhere [29]. Briefly, the animals were trained to sniff at two boxes equipped with absorbent paper strips that were impregnated with 20 µl of an odorant or the near odorless solvent signalling either that they contained a food reward (S+) or that they did not (S–). Opening of one of the boxes served as the operant response. 30 such trials (15 S+ and 15 S– trials in pseudorandomized order) using the same concentration of a given S+ were conducted per animal and condition.

Olfactory detection thresholds were determined by testing the animals' ability to discriminate between increasing dilutions of an odorant used as S+, and the near odorless solvent alone used as S–. Starting with a 100-fold liquid dilution, each stimulus was successively presented in 10-fold dilution steps until an animal failed to significantly discriminate the odorant from the solvent. Subsequently, an intermediate concentration (0.5 log units between the lowest concentration that was detected above chance and the first concentration that was not) was tested in order to determine the threshold value more exactly.

### Human test procedure

Olfactory sensitivity of the human subjects was assessed using a three-alternative forced choice procedure which has been described in detail elsewhere [39]. Briefly, subjects were presented with three randomly arranged bottles (250 ml high density polyethylene squeeze bottle equipped with a flip-up spout), two of which contained pure diluent and the third the stimulus (20 ml of liquid per bottle). In order to minimize adaptation effects, testing followed an ascending staircase procedure. Each bottle could be sampled twice per trial with an inter-stimulus interval of at least 5 s. Sampling duration was restricted to 1 s per presentation in order to minimize adaptation effects. Subjects were required to identify one of the three bottles as containing the stimulus. After the first decision, the bottles were rearranged and the subject allowed to sample again. If both choices with a given concentration were correct, this was provisionally recorded as the threshold dilution. However, if these had been preceded by one correct and one incorrect choice, the previous dilution (with a lower concentration of the odorant) was again tested, and if both choices were then correct this was taken as threshold. In this way, olfactory detection thresholds were determined for each subject. Testing was repeated in four more sessions as previous studies using the same method have shown that a subject’s performance may increase (that is, detection thresholds may decrease) with repeated testing and may reach a plateau by the third session [39]–[41]. The sessions were performed 1–3 days apart. Care was taken to systematically vary the order in which the odorants were presented across sessions.

For each odorant, a geometric dilution series in 14 steps was prepared, starting with a stem solution of 1:1,000, and progressing by a factor of 5. Stem dilutions were designated step 1, and subsequent dilutions step 2, 3, and so forth.

### Data analysis

For each individual animal, the percentage of correct choices from 40 (mice) and 30 (spider monkeys) trials per dilution step was calculated. With the mice, correct choices consisted both of licking in response to presentation of the S+ and not licking in response to the S–, and errors consisted of animals showing the reverse pattern of operant responses, that is: not licking in response to the S+ and licking in response to the S–. With the spider monkeys, correct choices consisted both of animals opening a box equipped with the S+ and failing to open a box equipped with the S-. Conversely, errors consisted of animals opening a box equipped with the S- or failing to open a box equipped with the S+. Significance levels were determined by calculating binomial z-scores corrected for continuity from the number of correct and false responses for each individual and condition. All tests were two-tailed and the alpha level was set at 0.05.

Post-hoc Shapiro-Wilk tests confirmed that the threshold values of the human subjects followed a normal distribution. They are therefore reported as group means ± SDs. Due to the low number of mice and spider monkeys tested, normality of data could not be verified. Therefore, threshold values for these two species are not reported as group means ± SDs but rather as individual data points, and between-species comparisons employed non-parametric tests. Comparisons of human group performance across sessions were made using the Friedman two-way analysis of variance. When ANOVA detected differences between sessions, this was then followed by pairwise Wilcoxon signed-rank tests for related samples to evaluate which sessions were responsible. Possible differences in sensitivity between male and female subjects and between species were assessed using the Mann-Whitney U-test for independent samples. Possible differences in sensitivity between odorants were assessed using the Wilcoxon signed-rank test for related samples. Correlations between across-odorant patterns of sensitivity of the three species were calculated using the Spearman rank-correlation test. Bonferroni corrections were used to counteract the problem of multiple comparisons wherever appropriate.

## Results

### CD-1 mice

**Figure 2:**
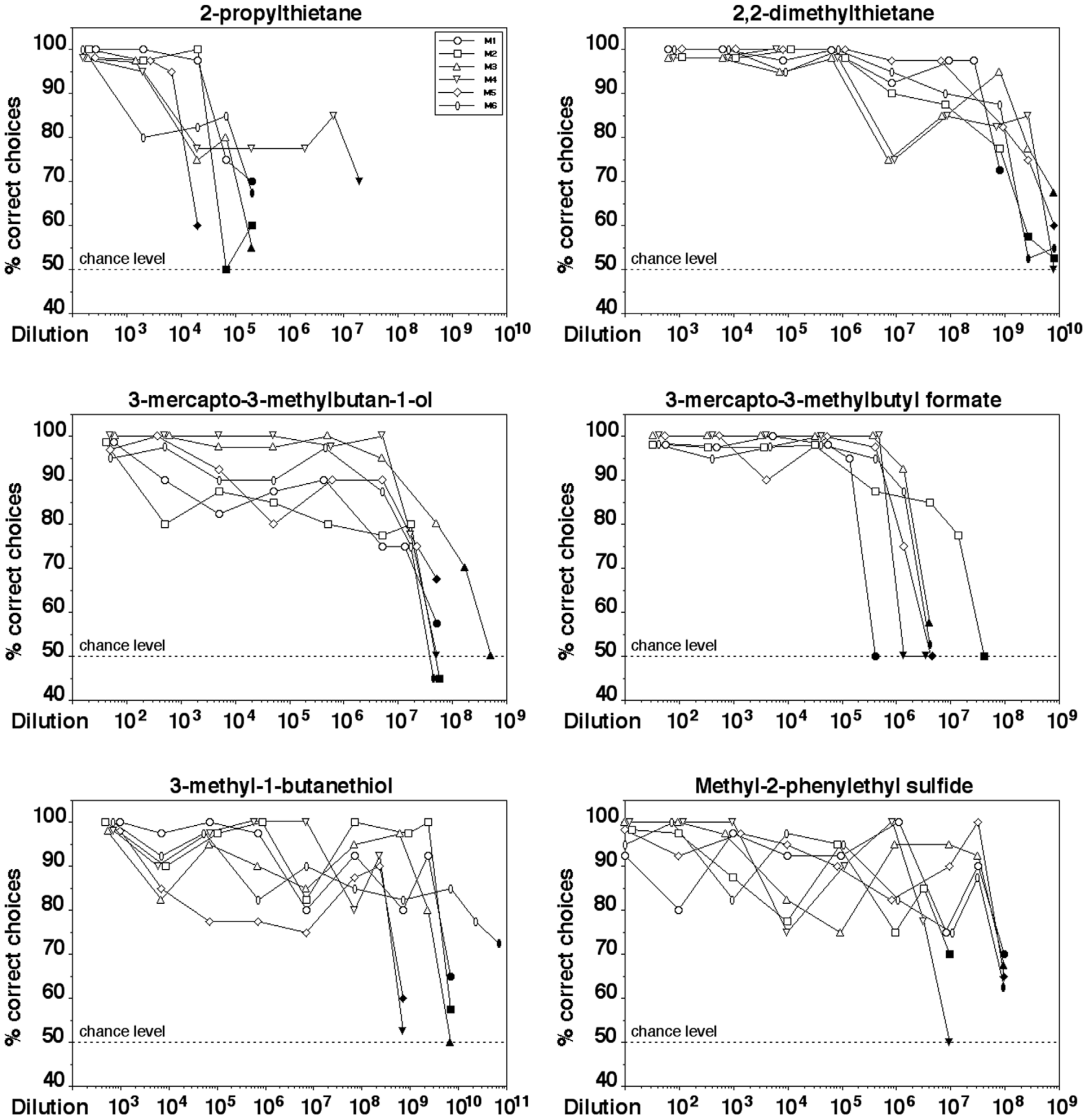
Performance of CD-1 mice in discriminating between various dilutions of a predator odorant and the solvent. Each data point represents the percentage of correct choices from a total of 40 decisions per individual animal. The six different symbols represent data from each of the six individual animals tested per odorant. Filled symbols indicate dilutions that were not discriminated significantly above chance level (binomial test, p>0.01).

Figure 2 shows the performance of the mice in discriminating between various dilutions of a given odorant and the solvent. All six animals significantly distinguished dilutions as low as 1:6.7•10^3^ 2-propylthietane, 1:2.7•10^8^ 2,2-dimethylthietane, 1:1.7•10^7^ 3-mercapto-3-methylbutan1-ol, 1:1.4•10^5^ 3-mercapto-3-methylbutyl formate, 1:2.3•10^8^ 3-methyl-1-butanethiol, and 1:3.2•10^6^ methyl-2-phenylethyl sulfide from the solvent (binomial test, p<0.01), with some individuals even scoring better. (Please note that the headspace above these dilutions was further diluted by a factor of 40 by the olfactometer used with the mice.)

The individual mice generally demonstrated similar detection threshold values with a given odorant and with three of the six odorants they differed only by a dilution factor of 10 (2,2-dimethylthietane and methyl-2-phenylethyl sulfide) or a factor of 3 (3-mercapto-3-methylbutan-1-ol) between the highest- and the lowest-scoring animal. With two of the odorants (3-mercapto-3-methylbutyl formate and 3-methyl-1-butanethiol) individual animals differed in their threshold values by a dilution factor of 100. The largest difference in sensitivity for a given odorant between individuals was a dilution factor of 1,000 and was found with 2-propylthietane.

Table 1 summarizes the threshold dilutions of the mice for the six odorants and shows various measures of corresponding gas phase concentrations [37] allowing readers to easily compare the data obtained in the present study to those reported by other authors using one of these convertible measures. In all cases, threshold values correspond to gas phase concentrations <0.1 ppm (parts per million). With the exception of 2-propylthietane, individual animals even reached threshold values as low as 1 ppt (parts per trillion) with all odorants tested.

**Table 1:** Olfactory detection threshold values in CD-1 mice for predator odorants, expressed in various measures of gas phase concentrations.

		liquid	gas phase concentration				
odorant	n	dilution	molec./cm^3^	ppm	log ppm	Mol/l	log Mol/l
2-propylthietane							
	1	1: 6.7•10^3^	7.5•10^11^	0.03	–1.52	1.3•10^−9^	–8.87
	1	1: 2.0•10^4^	2.5•10^11^	0.01	–2.00	4.5•10^−10^	–9.35
	3	1: 6.7•10^4^	7.5•10^10^	0.003	–2.52	1.3•10^−10^	–9.87
	1	1: 6.7•10^6^	7.5•10^8^	0.00003	–4.52	1.3•10^−12^	–11.87
2,2-dimethylthietane							
	1	1:2.7•10^8^	7.5•10^7^	0.000003	–5.52	1.3•10^−13^	–12.87
	2	1:8.1•10^8^	2.5•10^7^	0.000001	–6.00	4.5•10^−14^	–13.35
	3	1:2.7•10^9^	7.5•10^6^	0.0000003	–6.52	1.3•10^−14^	–13.87
3-mercapto-3-methylbutan-1-ol							
	5	1:1.7•10^7^	7.5•10^7^	0.000003	–5.52	1.3•10^−13^	–12.87
	1	1:5.1•10^7^	2.5•10^7^	0.000001	–6.00	4.5•10^−14^	–13.35
3-mercapto-3-methylbutyl formate							
	1	1:1.4•10^5^	7.5•10^9^	0.0003	–3.52	1.3•10^−11^	–10.87
	1	1:4.1•10^5^	2.5•10^9^	0.0001	–4.00	4.5•10^−12^	–11.35
	3	1:1.4•10^6^	7.5•10^8^	0.00003	–4.52	1.3•10^−12^	–11.87
	1	1:1.4•10^7^	7.5•10^7^	0.000003	–5.52	1.3•10^−13^	–12.87
3-methyl-1-butanethiol							
	2	1:2.3•10^8^	7.5•10^7^	0.000003	–5.52	1.3•10^−13^	–12.87
	3	1:2.3•10^9^	7.5•10^6^	0.0000003	–6.52	1.3•10^−14^	–13.87
	1	1:2.3•10^10^	7.5•10^5^	0.00000003	–7.52	1.3•10^−15^	–14.87
Methyl-2-phenylethyl sulfide							
	2	1:3.2•10^6^	7.5•10^7^	0.000003	–5.52	1.3•10^−13^	–12.87
	4	1:3.2•10^7^	7.5•10^6^	0.0000003	–6.52	1.3•10^−14^	–13.87

n indicates the number of animals.

No significant difference in olfactory sensitivity was found between mice that were 6 months old and mice that were 18 months old at the beginning of the study (Mann-Whitney U-test, p>0.05).

Across-odorant comparisons showed that the mice were significantly less sensitive to 2-propylthietane than to all five other predator odorants, including the structurally related 2,2-dimethylthietane (Wilcoxon, p<0.05). Similarly, the mice were significantly less sensitive to 3-mercapto-3-methylbutyl formate than to four of the five other predator odorants, including the structurally related 3-mercapto-3-methylbutan1-ol (Wilcoxon, p<0.05). The only other significant difference in sensitivity was found between 2,2-dimethylthietane and 3-mercapto-3-methylbutan1-ol, with the mice being more sensitive to the former than to the latter (Wilcoxon, p<0.05).

### Spider monkeys

**Figure 3:**
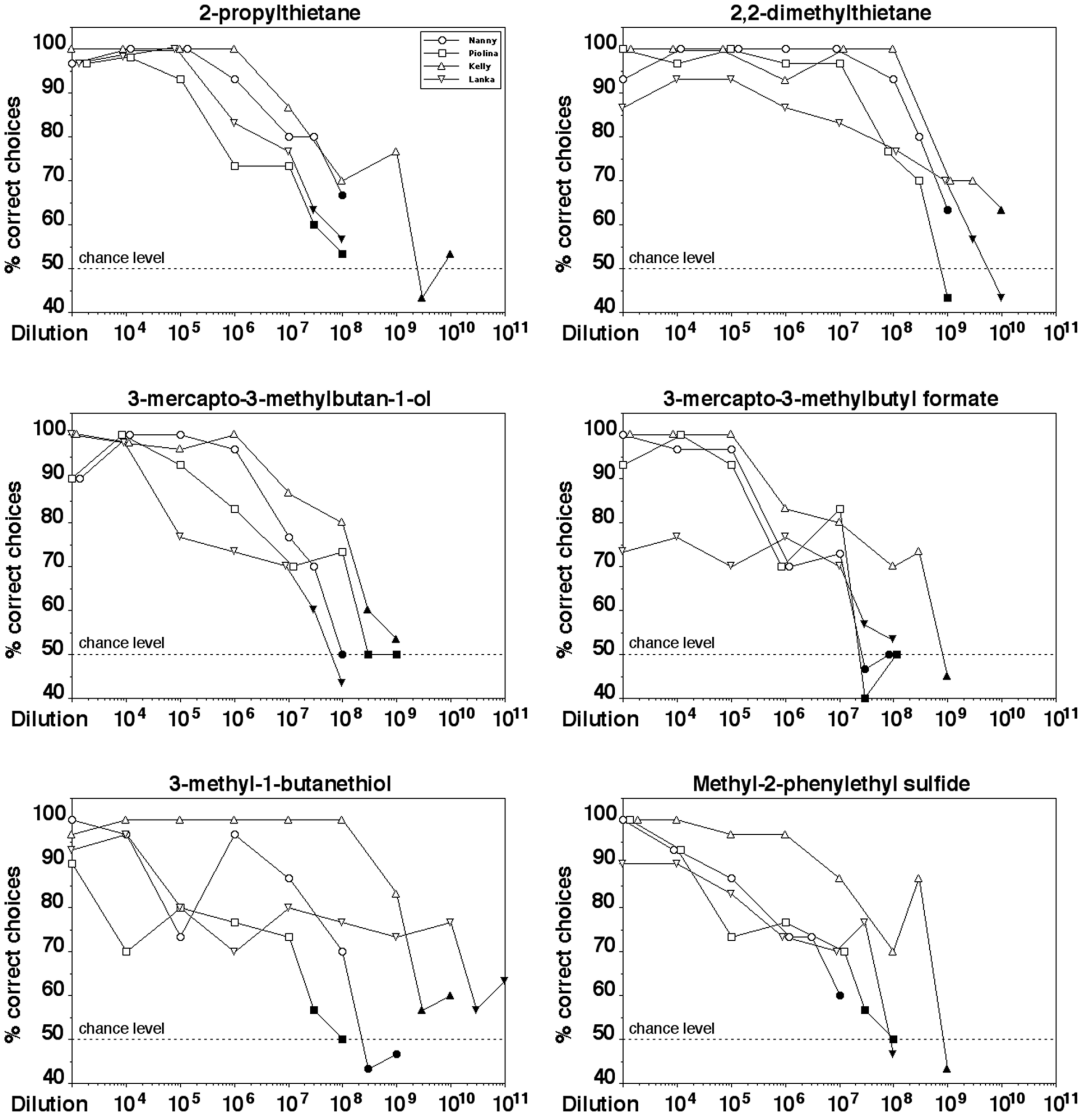
Performance of spider monkeys in discriminating between various dilutions of a predator odorant and the solvent. Each data point represents the percentage of correct choices from a total of 30 decisions per individual animal. The four different symbols represent data from each of the four individual animals tested per odorant. Filled symbols indicate dilutions that were not discriminated significantly above chance level (binomial test, p>0.01).

Figure 3 shows the performance of the spider monkeys in discriminating between various dilutions of a given odorant and the solvent. All four animals significantly distinguished dilutions as low as 1:10^7^ 2-propylthietane, 1:3•10^8^ 2,2-dimethylthietane, 1:10^7^ 3-mercapto-3-methylbutan-1-ol, 1:10^7^ 3-mercapto-3-methylbutyl formate, 1:10^7^ 3-methyl-1-butanethiol, and 1:3•10^6^ methyl-2-phenylethyl sulfide from the solvent (binomial test, p<0.05), with some individuals even scoring better.

The individual spider monkeys generally demonstrated similar detection threshold values with a given odorant and with three of the six odorants they differed only by a dilution factor of 10 (3-mercapto-3-methylbutan-1-ol and 2,2-dimethylthietane) or a factor of 33 (3-mercapto-3-methylbutyl formate) between the highest- and the lowest-scoring animal. With two of the odorants (2-propylthietane and methyl-2-phenylethyl sulfide) individual animals differed in their threshold values by a dilution factor of 100. The largest difference in sensitivity for a given odorant between individuals was a dilution factor of 1,000 and was found with 3-methyl-1-butanethiol.

Table 2 summarizes the threshold dilutions of the spider monkeys for the six odorants and shows various measures of corresponding gas phase concentrations [37]. In all cases, threshold values correspond to gas phase concentrations ≤1 ppb (parts per billion). With the exception of 2,2-dimethylthietane and 3-mercapto-3-methylbutan-1-ol, individual animals even reached threshold values as low as 10 ppt (parts per trillion) with all odorants tested.

**Table 2:** Olfactory detection threshold values in spider monkeys for predator odorants, expressed in various measures of gas phase concentrations.

		liquid	gas phase concentration				
odorant	n	dilution	molec./cm^3^	ppm	log ppm	Mol/l	log Mol/l
2-propylthietane							
	2	1: 10^7^	2.0•10^10^	0.00074	–3.13	3.3•10^-11^	–10.48
	1	1: 3•10^7^	6.7•10^9^	0.00025	–3.61	1.1•10^-11^	–10.95
	1	1: 10^9^	2.0•10^8^	0.0000074	–5.13	3.3•10^-13^	–12.48
2,2-dimethylthietane							
	2	1:3•10^8^	2.7•10^9^	0.0001	–4.00	4.5•10^-12^	–11.35
	1	1:10^9^	8.1•10^8^	0.00003	–4.52	1.3•10^-12^	–11.87
	1	1:3•10^9^	2.7•10^8^	0.00001	–5.00	4.5•10^-13^	–12.35
3-mercapto-3-methylbutan-1-ol							
	1	1:10^7^	5.1•10^9^	0.00019	–3.72	8.5•10^-12^	–11.07
	1	1:3•10^8^	1.7•10^9^	0.000063	–4.20	2.8•10^-12^	–11.55
	2	1:10^8^	5.1•10^8^	0.000019	–4.72	8.5•10^-13^	–12.07
3-mercapto-3-methylbutyl formate							
	3	1:10^7^	4.1•10^9^	0.00015	–3.82	6.8•10^-12^	–11.17
	1	1:3•10^8^	1.4•10^8^	0.0000052	–5.29	2.3•10^-13^	–12.63
3-methyl-1-butanethiol							
	1	1:10^7^	7.0•10^10^	0.0026	–2.59	1.2•10^-10^	–9.93
	1	1:10^8^	7.0•10^9^	0.00026	–3.59	1.2•10^-11^	–10.93
	1	1:10^9^	7.0•10^8^	0.000026	–4.59	1.2•10^-12^	–11.93
	1	1:10^10^	7.0•10^7^	0.0000026	–5.59	1.2•10^-13^	–12.93
Methyl-2-phenylethyl sulfide							
	1	1:3•10^6^	3.2•10^9^	0.00012	–3.93	5.3•10^-12^	–11.27
	1	1:10^7^	9.6•10^8^	0.000036	–4.45	1.6•10^-12^	–11.80
	1	1:3•10^7^	3.2•10^8^	0.000012	–4.93	5.3•10^-13^	–12.27
	1	1:3•10^8^	3.2•10^7^	0.0000012	–5.93	5.3•10^-14^	–13.27

n indicates the number of animals.

Across-odorant comparisons showed that the ranges of threshold values displayed by the spider monkeys overlapped with all six odorants. Accordingly, no significant differences in sensitivity between any of the six predator odorants were found (Wilcoxon, p>0.05).

### Human subjects

**Figure 4:**
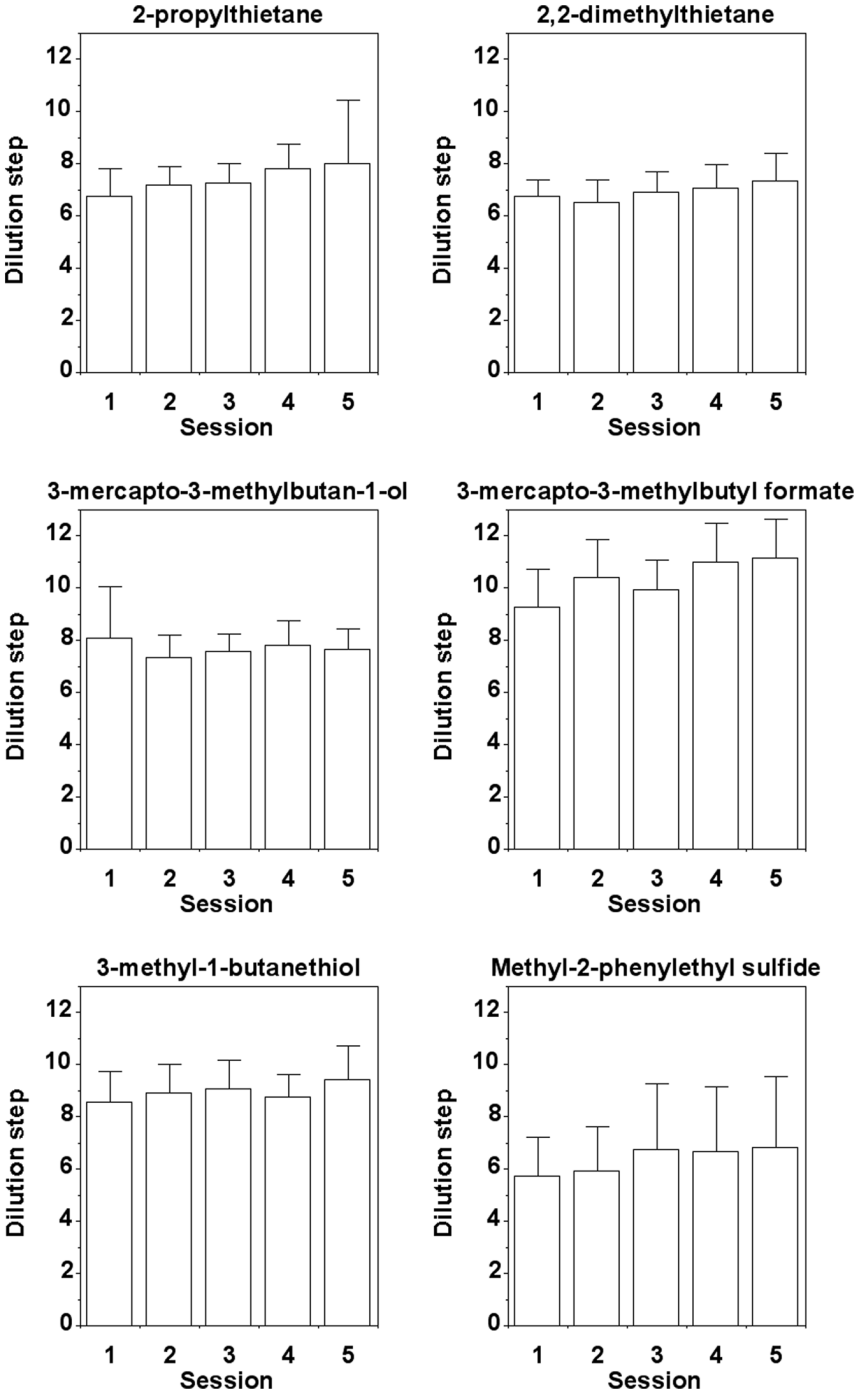
Olfactory detection thresholds of human subjects (n = 12) for the six predator odorants. Means and standard deviations for each of the five test sessions are given.

Figure 4 shows the mean detection thresholds of twelve human subjects for each of the six predator odorants tested across five sessions. Statistically significant differences between sessions were found with three of the six odorants (2-propylthietane, 2,2-dimethylthietane, and 3-mercapto-3-methylbutyl formate) (Friedman ANOVA, p<0.05). Significant between-session differences generally reflected an increase in group performance across sessions and only involved sessions 1 to 4 (Wilcoxon, p<0.05), but were not found between sessions 4 and 5 (Wilcoxon, p>0.05). This suggests that the group of subjects reached a plateau in performance during the last two sessions. Therefore, performance in session 5 is reported here as well as in Table 3 and used for between-species comparisons.

**Table 3:** Olfactory detection threshold values in human subjects for predator odorants, expressed in various measures of gas phase concentrations.

		dilution	gas phase concentration				
odorant		step	molec./cm^3^	ppm	log ppm	Mol/l	log Mol/l
2-propylthietane							
	H	6	6.4•10^10^	0.0024	–2.63	1.1•10^−10^	–9.97
	M	8.00	2.6•10^9^	0.000096	–4.02	4.3•10^−12^	–11.36
	L	14	1.6•10^5^	0.0000000059	–8.23	2.7•10^−16^	–15.58
2,2-dimethylthietane							
	H	6	2.6•10^11^	0.0096	–2.02	4.3•10^−10^	–9.36
	M	7.33	3.0•10^10^	0.0011	–2.95	5.0•10^−11^	–10.30
	L	9	2.1•10^9^	0.000078	–4.11	3.5•10^−12^	–11.46
3-mercapto-3-methylbutan-1-ol							
	H	6	1.6•10^10^	0.00059	–3.23	2.7•10^−11^	–10.58
	M	7.67	1.1•10^9^	0.000041	–4.39	1.8•10^−12^	–11.74
	L	9	1.3•10^8^	0.0000048	–5.32	2.2•10^−13^	–12.67
3-mercapto-3-methylbutyl formate							
	H	10	2.1•10^7^	0.00000078	–6.11	3.5•10^−14^	–13.46
	M	11.17	3.2•10^6^	0.00000012	–6.93	5.3•10^−15^	–14.27
	L	14	3.4•10^4^	0.0000000013	–8.90	5.6•10^−17^	–16.25
3-methyl-1-butanethiol							
	H	8	9.0•10^9^	0.00033	–3.48	1.5•10^−11^	–10.83
	M	9.42	9.1•10^8^	0.000034	–4.47	1.5•10^−12^	–11.82
	L	12	1.4•10^7^	0.00000052	–6.29	2.3•10^−14^	–13.63
Methyl-2-phenylethyl sulfide							
	H	4	7.7•10^10^	0.0029	–2.54	1.3•10^−10^	–9.89
	M	6.83	8.1•10^8^	0.000030	–4.52	1.3•10^−12^	–11.87
	L	14	4.3•10^4^	0.0000000016	–8.80	7.1•10^−17^	–16.15

n indicates the number of animals.

2-propylthietane was detected at dilution step 8.00±2.45 (mean±SD) and 2,2-dimethylthietane at dilution step 7.33±1.07, corresponding to a dilution of 1:7.7•10^7^ and 1:2.7•10^7^, respectively. 3-mercapto-3-methylbutan-1-ol was detected at dilution step 7.67±0.78 and 3-mercapto-3-methylbutyl formate at dilution step 11.17±1.47, corresponding to a dilution of 1:4.6•10^7^ and 1:1.3•10^10^, respectively. 3-methyl-1-butanethiol was detected at dilution step 9.42±1.31 and methyl-2-phenylethyl sulfide at dilution step 6.83±2.72, corresponding to a dilution of 1:7.7•10^8^ and 1:1.2•10^7^, respectively.

Table 3 summarizes the threshold dilutions of the human subjects for the six odorants and shows various measures of corresponding gas phase concentrations [37]. With all six odorants, mean threshold values correspond to gas phase concentrations ≤1 ppb (parts per billion). With four odorants (2-propylthietane, 3-mercapto-3-methylbutyl formate, 3-methyl-1-butanethiol, and methyl-2-phenylethyl sulfide) individual subjects even reached threshold values <1 ppt (parts per trillion).

With only one exception (2-propylthietane in session 4), no statistically significant differences in sensitivity between male and female subjects were found (Mann-Whitney U-test, p>0.05).

Across-odorant comparisons showed that the human subjects were significantly more sensitive to 3-mercapto-3-methylbutyl formate than to all five other predator odorants, including the structurally related 3-mercapto-3-methylbutan1-ol (Wilcoxon, p<0.05). The human subjects were significantly less sensitive to 2,2-dimethlythietane than to all other five odorants, including the structurally related 2-propylthietane (Wilcoxon, p<0.05). No significant differences in sensitivity between the other four predator odorants were found (Wilcoxon, p>0.05).

### Comparison between species

**Figure 5:**
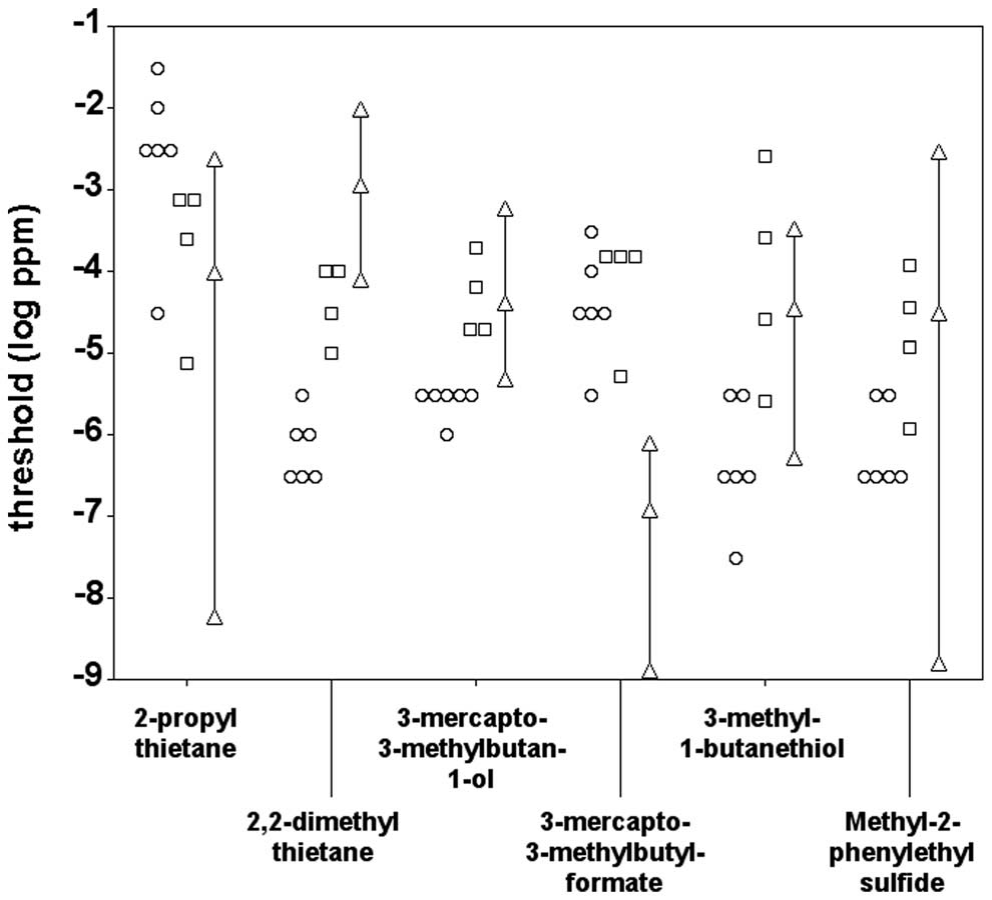
Comparison of the olfactory detection thresholds of the mice, the spider monkeys, and the human subjects for the six predator odorants. Data points of the mice (circles) and the spider monkeys (squares) represent threshold values of individual animals. Data points of the human subjects (triangles) represent the mean threshold value (from n = 12 subjects) and the highest and lowest individual threshold, respectively.

Figure 5 compares the olfactory detection threshold values of the three species for the six predator odorants tested. With four of the six odorants (2,2-dimethylthietane, 3-mercapto-3-methylbutan-1-ol, 3-methyl-1-butanethiol, and methyl-2-phenylethyl sulfide) the mice were signficantly more sensitive than the human subjects (Mann-Whitney U-test, p<0.05). However, the human subjects were significantly more sensitive than the mice with the remaining two odorants (2-propylthietane and 3-mercapto-3-methylbutyl formate) (Mann-Whitney U-test, p<0.05). Similarly, the mice were significantly more sensitive than the spider monkeys with four of the six odorants (2,2-dimethylthietane, 3-mercapto-3-methylbutan-1-ol, 3-methyl-1-butanethiol, and methyl-2-phenylethyl sulfide) (Mann-Whitney U-test, p<0.05). Mice and spider monkeys did not differ significantly in their sensitivity with the remaining two odorants (2-propylthietane and 3-mercapto-3-methylbutyl formate) (Mann-Whitney U-test, p>0.05), although it should be mentioned that all four spider monkeys displayed lower threshold values (that is: a higher sensitivity) with 2-propylthietane than five of the six mice (see Figure 5). Human subjects and spider monkeys did not differ significantly in their sensitivity with four of the six odorants (2-propylthietane, 3-mercapto-3-methylbutan-1-ol, 3-methyl-1-butanethiol, and methyl-2-phenylethyl sulfide) (Mann-Whitney U-test, p>0.05). The spider monkeys were significantly more sensitive than the human subjects with 2,2-dimethylthietane (Mann-Whitney U-test, p<0.05), and the human subjects were significantly more sensitive than the spider monkeys with 3-mercapto-3-methylbutyl formate (Mann-Whitney U-test, p<0.05). The across-odorant patterns of sensitivity did not correlate significantly between the three species (Spearman, humans vs. mice: r_s_ = +0.32; humans vs. spider monkeys: r_s_ = +0.19; mice vs. spider monkeys: r_s_ = +0.43; all p>0.05).

### Odor structure–activity relationships

Two of the six predator odorants (2-propylthietane and 2,2-dimethylthietane) share a thietane functional group, that is, a saturated four-membered ring with three carbon atoms and one sulfur atom, and only differ from each other in the alkyl rest attached to the thietane functional group (see Figure 1). Interestingly, both mice and human subjects differed significantly in their sensitivity for these two odorants (see Figure 5), suggesting that the alkyl rest attached to the thietane functional group markedly affected detectability of these odorants.

Two other of the six predator odorants (3-mercapto-3-methylbutan-1-ol and 3-mercapto-3-methylbutyl formate) share a thiol functional group (also known as a mercapto group) attached to a methylbutyl structure and only differ from eath other in the oxygen moiety at the far end of the molecule relative to the thiol group (see Figure 1). Here again, both mice and human subjects differed significantly in their sensitivity for these two odorants (see Figure 5), suggesting that the type of oxygen-containing functional group (alcohol or ester group) attached to the thiol functional group markedly affected detectability of these odorants.

## Discussion

The results of the present study demonstrate that mice have a well-developed olfactory sensitivity for sulfur-containing odorants known to be components of the odors of natural predators of the mouse. Further, they show that mice are significantly more sensitive than spider monkeys and human subjects with four of the six odorants tested, and that human subjects outperform mice with the remaining two odorants. Finally, the results show that molecular structural features such as the alkyl rest attached to a thietane or the oxygen-containing functional group attached to a thiol affected detectability in a species-specific manner.

A comparison of the threshold values obtained with the mice in the present study with those obtained in previous studies with other classes of odorants such as aliphatic aldehydes [14], carboxylic acids [15], aromatic aldehydes [16], alkylpyrazines [17], monoterpenes [18], “green odors” [19], and amino acids [20] shows that mice are particularly sensitive to the sulfur-containing predator odorants tested here. This should not be surprising given that these odorants have been shown to serve as kairomones, that is, as chemical signals informing prey species about the presence of predators [1], [2]. Previous studies also suggest that the behavioral relevance of odorants may affect an animal’s olfactory sensitivity. Rats, for example, have been reported to detect 2,4,5-trimethylthiazoline, a sulfur-containing fox odor component, at dramatically lower concentrations compared to non-predator odorants [42]. Non-prey species of the fox, in contrast, displayed considerably higher threshold values to this odorant than the rat [42].

A recent study which demonstrated that a mouse alarm pheromone shares structural similarity with sulfur-containing predator odorants such as 2-propylthietane and is detected by mouse olfactory sensory neurons at extraordinarily low concentrations lends further support to the idea that the behavioral relevance of odorants is likely to play an important role for an animal’s olfactory sensitivity [43]. Another recent study reported that mice possess a certain olfactory receptor (mOR244-3) which is selectively activated by thiols and other sulfur-containing odorants such as sulfides, polysulfides and thiocarbonyls, but not by odorants lacking a sulfur atom [44]. The presence of such a highly selective olfactory receptor might, at least partly, explain the high olfactory sensitivity of the mice found in the present study. Interestingly, humans possess a homologous receptor (named hOR4E2) which has not been deorphaned yet but should be expected to respond to the same sulfur-containing odorants as the mouse receptor mOR244-3 [45].

Similar to our findings with the mice, the threshold values obtained with the spider monkeys for the six predator odorants tested here are generally lower than (or in the lower range of) those reported for aliphatic esters [21], carboxylic acids [22], alcohols and aldehydes [23], monoterpenes [18], monoterpene alcohols [25], steroids [26], [27], alkylpyrazines [17], aromatic aldehydes [28], “green odors” [19], and amino acids [20]. Only the threshold values reported for thiols and indols [24] in the spider monkey are as low as those found in the present study. Similarly, the threshold values obtained with the human subjects for the six sulfur-containing predator odorants tested here are generally lower than (or in the lower range of) those reported for a variety of chemical classes lacking sulfur [46].

This raises the question as to why not only mice, but also spider monkeys and human subjects appear to be particularly sensitive to the predator odorants tested in the present study – despite the fact that the small-bodied mustelids, felids and canids known to be the source of these odorants are not exactly natural predators of humans. The only known mammalian predators of the spider monkey, a comparatively large-bodied New World primate, are the jaguar *(Panthera onca)* and the cougar *(Puma concolor)* [47], two felines whose urine, faeces and anal gland secretions have not yet been analyzed for the presence of the odorants used in the present study. (Please note that several of the predator odorants used here are thought to be species-specific or to be restricted to mustelids [4] and that only one occurrence of predation by a jaguar and a puma, respectively, on spider monkeys have been reported so far. Thus, it seems unlikely that spider monkeys are regularly exposed to any of the six predator odorants tested here, at least in the context of predator avoidance. Nevertheless, several studies demonstrated that nonhuman primates are able to distinguish between the faecal odors of natural predators and non-predators [48]–[50].)

This suggests that, at least in the case of the human subjects and the spider monkeys, it may not be the fact that the odorants tested here are predator odorants which underlies the high sensitivity observed in both species, but something else. Two alternative explanations should be considered: firstly, sulfur-containing odorants may generally be detected at low concentrations due to their physical properties. Secondly, the odorants may play an important role in a behavioral context other than predator avoidance. The first explanation is supported by psychophysical studies which have shown that human subjects generally detect sulfur-containing odorants at lower concentrations compared to structurally similar odorants in which the sulfur atom has been substituted for either an oxygen or a carbon atom [51], [52]. The molecular basis for the presumed importance of the sulfur atom for odor detection is that sulfur-containing volatiles are known to be strongly binding ligands for olfactory receptors due to their physicochemical properties [53]. In the case of the mouse olfactory receptor mOR244-3 which is selectively activated by thiols and other sulfur-containing odorants, for example, it has recently been demonstrated that the presence of copper ions plays a crucial role for the interaction of this receptor with its ligands [44].

The second explanation is supported by the fact that sulfur-containing odorants are not only characteristic for predator odors, but also for odors associated with putrefaction processes, that is, the microbial degradation of proteins [52], [54]. Thus, these odorants should be of particular importance to both human and nonhuman primates in the context of food selection as they need to avoid spoiled food due to their lack of specific physiological detoxification mechanisms [55]. Moreover, sulfur-containing odorants have been shown to be characteristic of breath odors [56] and may therefore play a role in the context of social communication. Several primate species have been reported to assess conspecifics by sniffing at the mouth [57] and the concentration of sulfur-containing odorants in oral breath odor has been found to change systematically as a function of the human menstrual cycle [58] and to be indicative of health status [59]. Which of these explanations, which are not mutually exclusive, may underlie our finding of a high olfactory sensitivity for sulfur-containing predator odorants in all three species requires further studies.

An across-species comparison of the threshold values obtained with the three species in the present study shows that the mice were significantly more sensitive than the human subjects and the spider monkeys with four of the six predator odorants. However, the human subjects were significantly more sensitive than the mice with the remaining two odorants. Human subjects and spider monkeys outperformed each other with one odorant each while no significant differences in sensitivity were found between these two species with the remaining four odorants. This is remarkable considering that the three species under investigation differ markedly from each other in the number of genes coding for olfactory receptors, with mice having ≈1,060 such genes [60], spider monkeys having ≈900 [61], and human subjects having only ≈390 [60]. This, in turn, suggests that it may not necessarily be the size, but perhaps the composition of the repertoire of functional olfactory receptor types that may determine a species’ olfactory sensitivity for a given odorant. This idea is supported by some previous studies which compared olfactory detection thresholds between species and also failed to find significant correlations between the size of olfactory receptor repertoires and olfactory sensitivity [16], [17], [21], [28].

Several studies have tried to link between-species differences in olfactory sensitivity to neuroanatomical properties such as the relative or the absolute size of the the olfactory bulbs [62]. However, although the relative size of the olfactory bulbs of the mouse (2.0% of total brain volume), the spider monkey (0.09%) and of human subjects (0.01%) [62] differ markedly from each other, the latter two species did not differ significantly in their sensitivity with four of six odorants tested here (and they outperformed each other with the remaining two odorants), and human subjects even outperformed the mouse with two of the six odorants (see Figure 5). Previous studies also led to ambiguous findings, with some of them supporting a positive correlation between the relative size of the olfactory bulbs and a species’ olfactory sensitivity [42] and some of them failing to do so [24], [27].

Similarly, although the three species under investigation differ markedly in the absolute size of their olfactory bulbs (mouse: 8.3 mm^3^, spider monkey: 90.4 mm^3^, human subjects 114 mm^3^ [62]), it is not exactly intuitive to explain the superior sensitivity of the mouse found with four of the six odorants tested here with having the smallest olfactory bulbs. Taken together, these comparisons lend further support to the notion that genetic features such as the number of functional olfactory receptor genes or neuroanatomical features such as the relative or the absolute size of the olfactory bulbs are poor predictors of a species’ olfactory sensitivity.

A final aspect of the present study is our finding that the alkyl rest attached to the two thietanes (2-propylthietane and 2,2-dimethylthietane) tested here, and that the type of oxygen-containing functional group attached to the two structurally related thiols (3-mercapto-3-methylbutan-1-ol and 3-mercapto-3-methylbutyl formate) significantly affected detectability in two of the three species under investigation.

Our finding that both the type of alkyl rest and the type of oxygen-containing functional group attached to an otherwise identical molecular backbone had a systematic effect on detectability in both mice and human subjects is in line with human studies that assessed the impact of these molecular structural features on olfactory detectability [63]. It is important to note, however, that the effect that a given type of oxygen-containing functional group had on olfactory sensitivity appears to be species-specific: the mice displayed significantly lower threshold values (and thus a higher sensitivity) with an alcohol group attached to a thiol structure compared to an ester group whereas the human subjects displayed the reverse pattern of sensitivity, and the spider monkeys did not differ in their sensitivity with both odorants (Figure 5). Similarly, the mice displayed lower threshold values with a dimethyl rest attached to a thietane structure compared to a propyl rest whereas the human subjects, here too, displayed the reverse pattern, and the spider monkeys, again, did not differ in their sensitivity with both odorants (Figure 5). One plausible explanation for the species-specificity of the effect that certain molecular structural features of the predator odorants tested here had on olfactory sensitivity is that the composition of functional olfactory receptor types may vary between species. This idea is supported by studies which reported that the olfactory sensitivity of human subjects, that is even within a given species, is associated with genetic variation in the genes coding for receptors involved in the perception of the odorants under investigation [64], [65].

Taken together, the olfactory detection threshold data reported here allow for the following conclusions:

1. Mice are significantly more sensitive than human subjects and spider monkeys with only four of the six predator odorants tested, and human subjects even outperform the mice with two of the odorants.

2. The number of functional olfactory receptor genes does not correlate positively with olfactory sensitivity for the odorants tested.

3. The relative and/or the absolute size of the olfactory bulbs does not correlate positively with olfactory sensitivity for the odorants tested.

4. With all three species tested, the threshold values obtained here are generally lower (or in the lower range of) those reported for other chemical classes tested previously, suggesting that sulfur-containing odorants may play a special role in olfaction.

5. In both mice and human subjects the type of alkyl rest attached to a thietane and the type of oxygen moiety attached to a thiol significantly affects olfactory sensitivity.

Further, the data may provide useful information for the choice of adequate stimulus concentrations in studies investigating the behavioral effects of predator odorants on prey and non-prey species.
